# Mind wandering in creative problem-solving: Relationships with divergent thinking and mental health

**DOI:** 10.1371/journal.pone.0231946

**Published:** 2020-04-23

**Authors:** Akina Yamaoka, Shintaro Yukawa

**Affiliations:** 1 Department of Human Welfare Counseling, Okinawa International University, Ginowan, Okinawa, Japan; 2 Faculty of Education, Hakuoh University, Oyama, Tochigi, Japan; Chiba Daigaku, JAPAN

## Abstract

Previous research has shown that mind wandering has both positive and negative effects. Mind wandering may improve creative problem solving; however, it could also lead to negative moods and poor mental health. It has also been shown that some forms of mental illness are positively related to creativity. However, the three factors of mind wandering, divergent thinking, and mental health have not been examined simultaneously, so it is possible that these relationships are manifested by spurious correlations. Therefore, we examined the relations among the three factors while controlling for each of their confounding effects. We asked 865 participants (458 men, 390 women, 17 unknown; M_age_ = 18.99 years, SD = 1.16) to complete a questionnaire measuring mind wandering traits, divergent thinking, and mental health measures including depressive symptoms and schizotypal personality. Multiple regression analysis showed that people who reported more depressive symptoms, schizotypal personality, and divergent thinking, were more likely to engage in mind-wandering. Our results indicated that frequency of mind wandering was linked to a risk of poorer mental health as well as to higher divergent thinking ability. In future research, we will examine the features of mind wandering related to divergent thinking and mental health by considering the contents of wandering thoughts and whether they are ruminative or not. We also need to examine whether the same results will be found when studying professionals in creative occupations, and when using different scoring methods in divergent thinking tests.

## Introduction

It is sometimes difficult for people to concentrate and stay in the here and now. Our minds tend to wander and generate thoughts that are unrelated to the current situation or task. This familiar mental phenomenon has been given various names such as daydreaming, thought intrusions, task irrelevant thought, spontaneous thought, stimulus independent thought, respondent thought, fantasy, task unrelated thought, task unrelated images, internally generated thought, self-generated thought, absentmindedness, zoning out, offline thought, undirected thought, unconsciousness thought, and “mind wandering” [1, 2]. After Smallwood and Schooler [3] suggested integrating these various lines of research into a conceptual body under the term mind wandering, the amount of research investigating this phenomenon increased. Most studies focused on the negative effects of mind wandering (e.g., [1,4]). For example, it has been shown that mind wandering prevents sustained attention to response tasks, which include major recognition tasks (e.g., [5,6]), reading tasks [1, 7], and lectures [8]. It has also been shown that mind wandering is associated with negative mood (e.g., [9–13]) and depression [14]. On the other hand, a few studies have focused on the positive aspects of mind wandering, such as its relationship with creativity [14–19] or planning for the future [4,20,21]. In particular, it is worth noting that past research on creativity tasks has shown that mind wandering that occurred during an incubation period improved scores on divergent thinking tests [16,18] and insight problem-solving [17]. Accordingly, we examined both the positive and negative effects of mind wandering, including its associations with mental illness and divergent thinking.

Previous research has shown that mind wandering is positively related to some mental illnesses. Huba, Aneshensel, and Singer [22] distinguished between a) positive constructive daydreaming/mind-wandering; b) guilty-dysphoric daydreaming/mind-wandering, and c) poor attentional control, suggesting the influence of mind wandering is different depending on its type. For example, it has been shown that when mind wandering takes the form of rumination, it is associated with increased health risks and worsening of mood [23], and only mind wandering without awareness was positively associated with depression [24]. In addition, some research suggests that wandering thoughts that were past- and other-related were associated with subsequent negative mood even if the thought content that followed was positive. On the other hand, future- and self-related wandering thoughts preceded improvements in mood, even when the subsequent thought content was negative [25]. Franklin et al. [26] noted that wandering thoughts that were rated as interesting were associated with an increase in positive mood. Although these studies suggest that the relationship between mind wandering and mood may be dependent on the content and form of mind wandering, many studies have found that the overall frequency of mind wandering is associated with worsening of mood [9,11–13,25]. Moreover, the relationship appears to be reciprocal: when a negative mood was induced, people’s minds also tended to wander more [10–13]. Based on this evidence, Ottaviani et al. [23] suggested that the relationship between mind wandering and depression is bi-directional. In fact, depressed patients showed a higher frequency of mind wandering than did healthy people [15]. Therefore, mind wandering is expected to be positively associated with proneness to depression.

Some studies have indicated that mind wandering is related to other mental health conditions such as schizophrenia [27]. For example, both mind wandering and schizophrenia are said to include a sense of being decoupled from the real world [27–30]. Cognitive disinhibition, which is a common trait of schizophrenia, is a condition similar to mind wandering [19]. In cognitive disinhibition, the cognitive filter—often called “latent inhibition”—becomes weak and the brain is flooded with often irrelevant information [31], which is much like mind wandering. In both cases, the influx of information seems to promote creativity. A study has also shown a significant relationship between creative achievement and reduced latent inhibition for people with high IQ [31]. Others have found a link between frequent mind wandering and higher creativity, which included results from divergent thinking tests [14,19] and the Creative Achievement Questionnaire [15].

Moreover, recent neuroscience research has shown that, on a brain structural level, higher creativity is consistently associated with higher gray matter density in the default mode network regions [32–34], which has been observed to become active during mind wandering [35–37]. These neurophysiological findings indicate that the association between creativity and mind wandering might even manifest at a trait level [38].

These studies showed that mind wandering is linked to both mental illness and creativity, and also showed the possibility of a positive relationship between mental illness and creativity. In fact, previous studies have often suggested a positive relationship between creativity and manifestations of mental illness, such as schizotypy, which is a tendency to have aberrant experiences that resemble milder versions of schizophrenia symptoms [39–41], especially positive schizotypy (odd perceptions and magical thinking) [42–45], bipolar disorder [41,46], and insomnia [47,48]. Although some articles reported that there were no significant relationships between creativity and mental illnesses such as attention deficit hyperactivity disorder, anxiety, social anxiety, negative affect, and depression [49,50], recently, Baas et al. [46] integrated these findings that positive schizotypy and bipolar disorder were positively related to creativity, whereas negative schizotypy (physical and social anhedonia), depressed mood, and anxiety were negatively related to it.

This, if creativity and mental illness are directly related, it is possible that the relationships between mind wandering and creativity, and between mind wandering and mental illness, are merely spurious correlations. Therefore, we conducted a survey of general college students and examined whether both mental health and creativity predict mind wandering while controlling for confounding effects of each of the other variables using multiple regression analysis. In our study, we used divergent thinking as an indicator of creativity. Divergent thinking is often used as an indicator of creativity, although some researchers have criticized it, arguing that it does not guarantee actual creativity [51]. We defined divergent thinking as involving a high potential for creative ideation and activation of associational networks to generate multiple unique solutions in a limited time period [52,53]. Additionally, we used depression tendency, which is commonly examined for its relationship to mind wandering and schizotypal personality, which has shown relationships with both mind wandering and creativity, as indicators of mental health.

## Method

### Participants and missing data procedure

Ethical approval for this study was obtained from the Faculty of Human Sciences’ Research Ethics Committee in University of Tsukuba.

We collected 890 participants’ questionnaires; however, we excluded data from 22 participants. This is because these participants answered on less than three scales, and the answers were dropped from the analysis; we also eliminated data from three participants because they responded using the same number across all scales, indicating that they may have answered carelessly. Therefore, we analyzed data from 865 respondents (458 men, 390 women, 17 unknown; M_age_ = 18.99 years, SD = 1.16) using SPSS Version 24.0 for Microsoft Windows (IBM company). We employed a single imputation method using the Expectation-Maximization Method and estimated the few missing data points. A power analysis showed that a sample size ≥ 92 is required to detect a medium-sized effect (*f*^*2*^ = 0.15; *α* = 0.05; 1-*β* = 0.8). Thus, the sample size in this study was confirmed to be was sufficient for the effect size.

### Procedure and ethical considerations

We conducted a survey by recruiting participants from 13 classes at the University of Tsukuba. A questionnaire was distributed to the students after the class and we announced that the purpose of this survey was to examine the relationships between creativity, mental health, and attention, and explained the following ethical considerations: (a) this questionnaire is not related to your grade evaluation, (b) even if you do not participate in this survey or withdraw participation, you will never be at a disadvantage, (c) since the survey is done anonymously, your privacy will be strictly maintained and the results of the survey will be used only for research, and (d) with your answer, it is assumed that you agree to cooperate with the survey. This information was also printed on the face sheet of the questionnaire. We also told them not to open the questionnaire until they received our instruction.

We instructed them that, “If you do not agree, do not answer the questionnaire and if you agree to cooperate with the survey, please open the face sheet of the questionnaire.” First, we conducted the divergent thinking test at the same time because the divergent thinking test has a time limit for answering of three minutes for each question. After the divergent thinking test, we told them to move on and complete the next questionnaire at their own pace. Our survey took about 15 minutes and participants did not receive any reward.

### Questionnaires

We used the Unusual Uses Test (UUT) [54] as an indicator of divergent thinking. In this test, participants are required to list as many unusual uses for common things as they can within a time limit. First, we conducted the test using the object “brick” with a time limit of 30 seconds as practice. After that, “can” and “socks” were used with a time limit of three minutes each. Answers were scored according to three aspects of divergent thinking: fluency, flexibility, and originality. Fluency indicates the ability to generate a significant number of answers, so the number of answers was scored as fluency [54]. Flexibility is the ability to propose various perspectives, so the number of categories that could be assigned to answers was scored as flexibility [54]. For example, if a participant answered, “dust box” and “jewelry box,” as unusual uses of a can, “box” became a category. Each category was worth one point. Lastly, originality is the ability to produce unique ideas. Thus, each answer was compared to the answers from other respondents. If an answer was provided by less than 5% of respondents, one point was given and if less than 1%, two points were given. In accordance with these instructions and the example, the first author calculated fluency and originality, and an evaluator who belongs to the faculty of psychology scored flexibility.

We then measured participants’ depressive symptom levels using the Japanese version of The Center for Epidemiologic Studies Depression Scale (CES-D) [55,56], which is a 20-item list using a four-point Likert scale for each item (“A: rarely or none of the time” “B: some or a little of the time,” “C: occasionally or a moderate amount of time,” and “D: most or all of the time”). We modified the instructions of the scale to measure students’ daily tendencies toward depression, as opposed to those in the past week (“We will ask about your daily mental and physical condition”). We calculated total scores of the CES-D by coding A = 0, B = 1, C = 2, and D = 3 (items 4, 8, 12, and 16 were reversed), so the possible score range was from 0 to 60 points. Cronbach’s α was .88 in our sample.

We measured schizotypal personality using the Japanese version of the Schizotypal Personality Questionnaire Brief [57,58], which consists of 22 items. Participants are asked to select “Yes” (one point) or “No” (zero points). We calculated the total schizotypal personality score and the possible score range was 0 to 22. Cronbach’s α was .75 in our sample.

Finally, we measured participants’ tendency toward mind wandering using the Japanese version of the Mind-Wandering Questionnaire [59,60]. The test includes five items and a six-point Likert scale (from “1: almost never” to “6: almost always”). We calculated the average score of the mind wandering trait. Although Cronbach’s α was lower than the usually accepted threshold (α = .69 in our sample), this seemed to be so because a ceiling effect occurred in the fifth item. However, considering content validity, we used all items for analysis.

## Results

Table 1 shows the means and standard deviations of each variable and Table 2 shows Pearson’s correlation coefficients among all variables. Significant positive relationships between mind wandering and each subscale of divergent thinking (fluency: *r* = .14, *p* < .001; flexibility: *r* = .16, *p* < .001; originality: *r* = .12, *p* < .001) were found, along with positive relationships between mind wandering and mental health (schizotypal personality: *r* = .23, *p* < .001; depressive symptoms: *r* = .33, *p* < .001). In addition, although the correlation coefficients were small, there were significant positive relationships between subscales of divergent thinking and schizotypal personality (fluency: *r* = .17, *p* < .001; flexibility: *r* = .17, *p* < .001; originality: *r* = .17, *p* < .001), and depressive symptoms (fluency: *r* = .07, *p* < .05; flexibility: *r* = .07, *p* < .05). Except for the correlation between divergent thinking and depressive symptoms, *p*-values were lower than the alpha level adjusted by Bonferroni correction (0.05/21 = 0.002).

**Table 1:** Means and standard deviations of variables.

	*M*	*SD*	*Skewness*	*Kurtosis*
Mind wandering	4.07	0.71	-0.52	1.92
Divergent thinking	0.00	2.91	1.14	2.45
Fluency	5.15	2.57	1.21	2.63
Flexibility	4.20	1.82	0.71	0.87
Originality	6.76	4.47	1.68	5.33
Schizotypal personality	8.87	4.07	0.08	-0.38
Depressive symptoms	18.49	8.95	0.62	0.18

Means of schizotypal personality and depressive symptoms are total scores of each scale. Possible score ranges are as follows: schizotypal personality– 0 to 22 points and depressive symptoms– 0 to 60 points.

**Table 2:** Correlations between variables of mind wandering, divergent thinking, and mental health.

	2	3	4	5	6	7
1. Mind wandering	.15***	.14***	.16***	.12***	.23***	.33***
2. Divergent thinking		.99***	.96***	.97***	.17***	.07
3. Fluency			.92***	.95***	.17***	.07*
4. Flexibility				.87***	.17***	.07*
5. Originality					.17***	.05
6. Schizotypal personality						.48***
7. Depressive symptoms						

*** *p* < .001,

**p < .01,

*p < .05

Next, we conducted multiple regression analysis (Table 3). To avoid multicollinearity, we used the variable “divergent thinking,” which comprised total scores of standardized fluency, standardized flexibility, and standardized originality. In the multiple regression analysis, we used mind wandering as a dependent variable and divergent thinking, schizotypal personality, and depressive symptoms as independent variables. We also controlled for age and sex. Expect for control variables, all independent variables positively predicted mind wandering (*βs* = .08–.28).

**Table 3:** Results of multiple regression analysis using mind wandering, divergent thinking, and mental health.

	Mind wandering
	*β*
Sex	-.04
Age	.01
Divergent thinking	.11***
Schizotypal personality	.08*
Depressive symptoms	.28***
*F*	25.31***
*Adjusted R*^*2*^	.12

* *p* < .05,

*** *p* < .001

## Discussion

In this study, we examined whether mental health and divergent thinking each predict mind wandering while controlling for confounding effects of each of the other variables. The multiple regression analysis results showed that people who have higher levels of depressive symptoms and schizotypal personality tended to report a higher frequency of mind wandering. The results, which showed a relationship between depression and mind wandering, were consistent with those of previous studies that demonstrated that frequency of mind wandering is related to negative mood (e.g., [9,25,26]) and depression [14]. According to previous research [23], a two-way causal relationship can be considered in which people with high depression tend to experience mind-wandering with negative content or ruminative mind-wandering, and their depression tends to increase.

Schizotypal personality was positively correlated with depression tendency, but schizotypal personality also predicted mind wandering independently, even after controlling for the effects of depression tendency. This supports previous research [27], and is likely attributable to the fact that mind wandering and schizotypal personality share some similar aspects in that participants are said to have a sense of being decoupled from the real world [27–30] and there is a great influx of information in both mind wandering and cognitive disinhibition, which is a common factor with schizophrenia [19]. Thus, our study measured depression and schizotypal personality as indicators of mental health, but these variables independently predicted mind wandering and their associations have different causes.

The regression coefficient between mind wandering and divergent thinking was also statistically significant, which is consistent with previous research (e.g., [15]). An association between creativity and mind wandering has been found, and this included studies examining the relation with the default mode network and conducted on the brain structure level [32–34, 38]. Our findings supported these previous studies. Furthermore, although our research does not show a causal relation, this positive relation could have occurred because people who engage in mind wandering in everyday life have an abundance of information in their minds, which promotes divergent thinking, as with people high in cognitive disinhibition [31,61]. In fact, previous research [16–18] has showed that when mind wandering occurred during an incubation period, creative problem solving was enhanced.

Therefore, in further research, we should examine whether, when mind wandering has occurred, creativity would be improved and negative affect would also be generated. Our results indicated the possibility that, although state level mind wandering can improve creativity, it may also lead to poor mental health.

### Limitations

Overall, our results seem to indicate the possibility that more mind wandering is accompanied by greater divergent thinking and decreased mental health. However, our study has several limitations. First, according to previous research, although the overall trait of mind wandering is positively related to depression, it is possible that only past- and other-related thoughts [25], ruminating thoughts [22], or mind wandering without awareness [24] are positively related to depression. Moreover, Agnoli et al. [15] showed that deliberate mind wandering is positively related to creativity while spontaneous mind wandering is negatively related to it. Although they used the Creative Achievement Questionnaire [52] as an indicator of creativity, not as an indicator of divergent thinking, there is a possibility that the association with divergent thinking differs depending on the intentionality behind the mind wandering. Second, the size of the regression coefficient between mind wandering and divergent thinking was small. A third variable such as intelligence or working memory capacity may affect the relationship between them [19]. In fact, previous research on cognitive disinhibition has shown that people low in latent inhibition showed high creative achievement when they also had high intelligence [31]. They hypothesized that high intelligence enables a person to process and manipulate unfiltered stimuli that result from low latent inhibition, thereby increasing the odds of original recombinant ideation. Thus, we need to account for other variables in future research. Third, we need to examine whether the same positive relationship between mind wandering and divergent thinking is found when using other types of creativity tests. Although the UUT is a common test of divergent thinking and is sometimes used as a creativity test, we should examine other types of creativity tests such as insight tests that require mainly convergent thinking. Previous research has shown that mindfulness is positively associated with insight problem-solving [62]. Thus, it is possible that mind wandering is positively related to performance on divergent thinking tests and negatively related to performance on convergent thinking tests such as insight problem-solving tests. Moreover, we need to examine whether different methods of scoring divergent thinking would lead to different results because previous research showed that relationships between psychopathology measures and divergent thinking depend on how divergent thinking tests are scored [63]. In addition, professional artists, musicians, writers, or other professionals in creative occupations need to be surveyed to determine whether they showed results similar to those of the students in our study.

## Conclusion

Although there are several limitations to our study and more detailed surveys are needed, we showed fundamental and overall relationships between mind wandering, divergent thinking, and mental health, while controlling for the possibility of spurious correlations. In the future, we will examine these relationships more elaborately, considering the creativity test type, participants’ attributes, and the specific nature of mind wandering.

## Supporting information

S1 Dataset Means and standard deviations of variables. (XLSX)

S2 Dataset Correlations between variables of mind wandering, divergent thinking, and psychopathology spectrum. (XLSX)

S3 Dataset Results of multiple regression analysis using mind wandering, divergent thinking, and psychopathology spectrum. (XLSX)

S1 File Data set. (XLSX)
